# Single bolus versus split dose gadolinium administration in extra-cellular volume calculation at 3 Tesla

**DOI:** 10.1186/s12968-015-0112-6

**Published:** 2015-01-31

**Authors:** Adam K McDiarmid, Peter P Swoboda, Bara Erhayiem, David P Ripley, Ananth Kidambi, David A Broadbent, David M Higgins, John P Greenwood, Sven Plein

**Affiliations:** Multidisciplinary Cardiovascular Research Centre (MCRC) & Leeds Institute of Cardiovascular and Metabolic Medicine, University of Leeds, Leeds, UK; Philips Healthcare, Philips Centre, Guildford Business Park, Guildford, UK

## Abstract

**Background:**

Diffuse myocardial fibrosis may be quantified with cardiovascular magnetic resonance (CMR) by calculating extra-cellular volume (ECV) from native and post-contrast T1 values. Accurate ECV calculation is dependent upon the contrast agent having reached equilibrium within tissue compartments. Previous studies have used infusion or single bolus injections of contrast to calculate ECV. In clinical practice however, split dose contrast injection is commonly used as part of stress/rest perfusion studies. In this study we sought to assess the effects of split dose versus single bolus contrast administration on ECV calculation.

**Methods:**

Ten healthy volunteers and five patients ( 4 ischaemic heart disease, 1 hypertrophic cardiomyopathy) were studied on a 3.0 Tesla (Philips Achieva TX) MR system and underwent two (patients) or three (volunteers) separate CMR studies over a mean of 12 and 30 days respectively. Volunteers underwent one single bolus contrast study (Gadovist 0.15mmol/kg). In two further studies, contrast was given in two boluses (0.075mmol/kg per bolus) as part of a clinical adenosine stress/rest perfusion protocol, boluses were separated by 12 minutes. Patients underwent one bolus and one stress perfusion study only. T1 maps were acquired pre contrast and 15 minutes following the single bolus or second contrast injection.

**Results:**

ECV agreed between bolus and split dose contrast administration (coefficient of variability 5.04%, bias 0.009, 95% CI −3.754 to 3.772, r^2^ = 0.973, p = 0.001)). Inter-study agreement with split dose administration was good (coefficient of variability, 5.67%, bias −0.018, 95% CI −4.045 to 4.009, r^2^ = 0.766, p > 0.001).

**Conclusion:**

ECV quantification using split dose contrast administration is reproducible and agrees well with previously validated methods in healthy volunteers, as well as abnormal and remote myocardium in patients. This suggests that clinical perfusion CMR studies may incorporate assessment of tissue composition by ECV based on T1 mapping.

## Background

Expansion and composition change of the myocardial extra-cellular matrix (ECM) is seen in a range of myocardial diseases and correlates with measures of systolic and diastolic function [1-5]. Cardiovascular magnetic resonance (CMR) late gadolinium enhancement (LGE) imaging is well suited for the detection of focal myocardial scar that characterises a number of disease processes [6,7]. However this technique relies upon the presence of healthy myocardium to detect scar, and as a result is limited in the detection of diffuse myocardial disease processes where global myocardial ECM expansion occurs.

Longitudinal relaxation time (T1) mapping allows quantitative characterisation of the myocardium, thereby enabling detection of diffuse myocardial disease processes that has previously required cardiac biopsy [8,9]. Furthermore, the ability to accurately define myocardial composition allows for the detection of sub-clinical disease states and may enable the effects of intervention on tissue composition to be determined non-invasively [10].

T1 measurement before and after the administration of an extra-cellular contrast agent allows the relative volumes of the intra-cellular and extra-cellular components of myocardial tissue to be quantified as long as equilibrium between the extra-cellular compartments (interstitium and blood) has been reached. Equivalence of primed slow intra-venous infusion and bolus only administration of contrast agent has been demonstrated previously [11,12]. However, stress perfusion imaging is an expanding area of CMR [13] now included in international practice guidelines [14]. During stress perfusion CMR studies, contrast agent delivery is split between rest and stress imaging. Integrating T1 mapping and ECV calculation in such a clinical protocol requires knowledge of the effects of split contrast injection on the derived measurements. Therefore we aimed to determine the effects of split versus single bolus contrast administration on ECV, and to assess the inter-study variability of ECV measured on split contrast administration CMR studies.

## Methods

The research protocol was approved by the local ethics committee and all subjects gave written informed consent. All studies were performed at a single centre equipped with a 3 T MRI scanner (Achieva TX, Philips Healthcare, Best, The Netherlands) using RF shimming and a 32-channel cardiac phased array receiver coil.

### Volunteer scanning

A total of ten healthy volunteers were recruited to undergo CMR. Subjects were excluded if they had a history of cardiac disease, hypertension, renal impairment, diabetes or contra-indication to CMR.

All subjects underwent a total of three CMR studies on separate days.

Study 1. Single bolus: In one CMR study, the contrast agent (Gadovist, Bayer Schering Pharma, Berlin-Wedding, Germany) was administered as a single bolus (0.15 mmol/kg) with post-contrast T1 mapping acquired 15 minutes later.

Study 2. Split dose: Contrast was administered as a split doses (0.075 mmol/kg twice) as part of an adenosine stress perfusion protocol. For stress perfusion, intra-venous adenosine was administered at 140 mcg/kg/min, via an intra-venous cannula sited in the ante-cubital fossa, for a minimum of three minutes and until an appropriate haemodynamic response had occurred. Contrast agent was delivered at a dose of 0.075 mmol/kg at peak haemodynamic stress. For rest perfusion, the same contrast injection regime was repeated twelve minutes later. A total Gadovist dose of 0.15 mmol/kg was administered. Post-contrast T1 mapping was performed 15 minutes after the second contrast administration.

Study 3. Split dose: Split dose stress perfusion CMR study as 2.

### Patient scanning

A total of five patients that had undergone a clinically indicated, non-urgent, adenosine stress perfusion CMR were recruited if they exhibited an area of enhancement on LGE imaging on the clinical study, which included native T1 map and 15 minute post contrast T1 map as per our local protocol. Patients were then recalled for one single bolus CMR study on a separate day in keeping with the above protocols.

### CMR protocol

In each study, the cardiac long-axis was located as per standard practice using balanced steady-state free precession pulse (bSSFP) cine images. Cine imaging was performed using a contiguous stack of parallel short-axis slices covering the whole left ventricle (LV), with a bSSFP pulse sequence (echo time (TE) 1.3 ms; repetition time (TR) 2.6 ms; flip angle 40°, spatial resolution 1.6 × 2.0 × 10 mm, 40 phases per cardiac cycle).

For all studies, contrast was delivered via a peripheral cannula, followed by a 20 ml saline flush delivered by automated injector (Medrad Inc, Warrendale, Pennsylvania, USA) at 5 ml/second.

Native and 15 minute post-contrast data for T1 value estimation were obtained using breath-held Modified Look-Locker Inversion recovery (MOLLI) acquisition [10,15,16]. Images were acquired in the central slice of a ‘3 of 5’ approach [17]. An ECG triggered 5b(3 s)3b MOLLI balanced turbo gradient recalled echo (GRE) acquisition method was used (voxel size 1.98×1.98×10 mm^3^ (reconstructed to 1.25×1.25 mm), single-shot, sensitivity encoding (SENSE) factor 2, trigger delay set for end-diastole, flip angle 35°, acquisition duration per image 170-185 ms (dependent upon FOV) a range of inversion times are calculated by the system in order to provide good sampling of T1 recovery.

Perfusion imaging acquisition used a spoiled turbo GRE sequence (echo time (TE) 2.8 ms; repetition time (TR) 1.28 ms; flip angle 15°, acquired spatial resolution 2.42×2.42 × 10 mm) in three 10 mm short axis slices with a 148×148 matrix, FOV 300–420, sensitivity encoding factor 2.4, half scan factor of 0.65 and a saturation pre-pulse delay of 80 ms.

LGE imaging was performed at 7–10 minutes following final contrast dose (inversion recovery-prepared T1 weighted gradient echo, inversion time according to Look-Locker scout, TR/TE/flip angle 3.7/2.0/25 degrees, spatial resolution 1.54 × 1.75 × 10 mm).

### Image analysis

Study images were saved as Digital Imaging and Communications in Medicine (DICOM) format. T1 values were calculated from source images using manual motion correction on CMR42 (Circle Cardiovascular Imaging Inc, Calgary, Alberta, Canada). Mis-registration was avoided by visually comparing left and right ventricular anatomical features (papillary muscles, trabeculations) any mis-registered images were discarded. In volunteers a narrow region of interest (ROI) in the infero-septum of the mid-ventricular slice was drawn as per Puntmann [18] in an effort to minimise potential artefact induced by epicardial cardiac vessels in the anterior and lateral walls. In patient studies two separate ROIs were drawn sampling:

1. the area displaying visual enhancement on the LGE acquisition; matched using standard image planning techniques and left and right ventricular anatomical features.
2. remote myocardium (preferentially the infero-septum as in volunteer studies).

The blood pool contour was drawn in the centre of the LV cavity on the same slice away from any papillary muscle. Signal intensity was measured from each MOLLI source image and T1 estimated based on the mean signal from myocardial and blood pool ROIs. ECV was calculated using the formula:

$$ ECV=\left(1 - Hct\right)\frac{R1\left(myo\ pre\right)-R1\left(myo\  post\right)}{R1\left( blood\ pre\right)-R1\left( blood\  post\right)} Where\ R 1= 1/T 1 $$

### Statistical analysis

Statistical analysis was performed using IBM SPSS® Statistics 21.0 (IBM Corp., Armonk, NY). Unless otherwise stated the results are presented as mean ± standard deviation (SD). Reproducibility and agreement was assessed by coefficient of variation and Bland Altman plot. Normality of distribution was determined with Kolmogarov-Smirnov testing, normality was assumed with a value of >0.2. Correlation was assessed with Pearson’s correlation coefficient.

## Results

Demographic characteristics of the study volunteers are as presented in Table 1. The mean age of volunteers was 26.6 yrs ± 2.8, 7 of 10 volunteers were men. Body surface area (BSA) corrected LVEDV (101 ± 12 ml/m^2^), LV mass (52 ± 7 g/m^2^) and ejection fraction (57% ± 2) were normal. All volunteers had normal right ventricular function and demonstrated no hyper-enhancement on late gadolinium enhancement images. All volunteers had an appropriate response to adenosine, with mean resting heart rate of 61.4 ± 5.2 beats per minute that increased on stress perfusion by 22.6 ± 7.6 beats per minute. Stress perfusion images were assessed qualitatively and no perfusion defects were identified. Split dose administration contrast agent doses were separated by 12.0 ± 3.7 minutes.

**Table 1:** **Subject characteristics**

	**Healthy volunteers (n = 10)**	**Patients (n = 5)**
**Age**	26.6 ± 2.8	59 ± 13.3
**Gender (M:F)**	7 : 3	4 : 1
**Underlying Cardiac Disease**		Ischaemic Heart Disease 4 Hypertrophic Cardiomyopathy 1
**Mean Rest Systolic Blood Pressure (mmHg)**	111 ± 7	131 ± 19
**Mean Rest Diastolic Blood Pressure (mmHg)**	55 ± 6	71 ± 10
**Mean Rest Heart Rate (bpm)**	61.4 ± 5.2	63 ± 19
**Mean Stress Heart Rate Increase**	22.6 ± 7.6	86 ± 16
**BSA indexed LVEDV (ml/kg/m** ^**2**^ **)**	101.3 ± 12.3	96.2 ± 35.8
**BSA indexed LV Mass (g/kg/m** ^**2**^ **)**	51.5 ± 7.1	64.3 ± 20.3
**LV EF %**	57 ± 2	41.7 ± 13.2
**RV EF %**	54 ± 2	48.8 ± 5.9

Patient characteristics are as displayed in Table 1. Mean patient age was 59 yrs ± 13.3, 4 of 5 patients were men, body surface area (BSA) corrected LVEDV (96 ± 36 ml/m^2^), LV mass (64 ± 20/m^2^) and ejection fraction (42% ± 13). Patient pathologies were: 4 chronic ischaemic heart disease with established myocardial infarction; 1 hypertrophic cardiomyopathy. All patients had extensive LGE, and no inducible perfusion defects on stress perfusion imaging outwith the area of LGE.

29 of 30 sets of volunteer mid-ventricular short axis MOLLI acquisitions were available for final analysis; in one MOLLI an acquisition error prohibited analysis. 10 of 10 patient MOLLI acquisitions were suitable for analysis.

**Figure 1:**
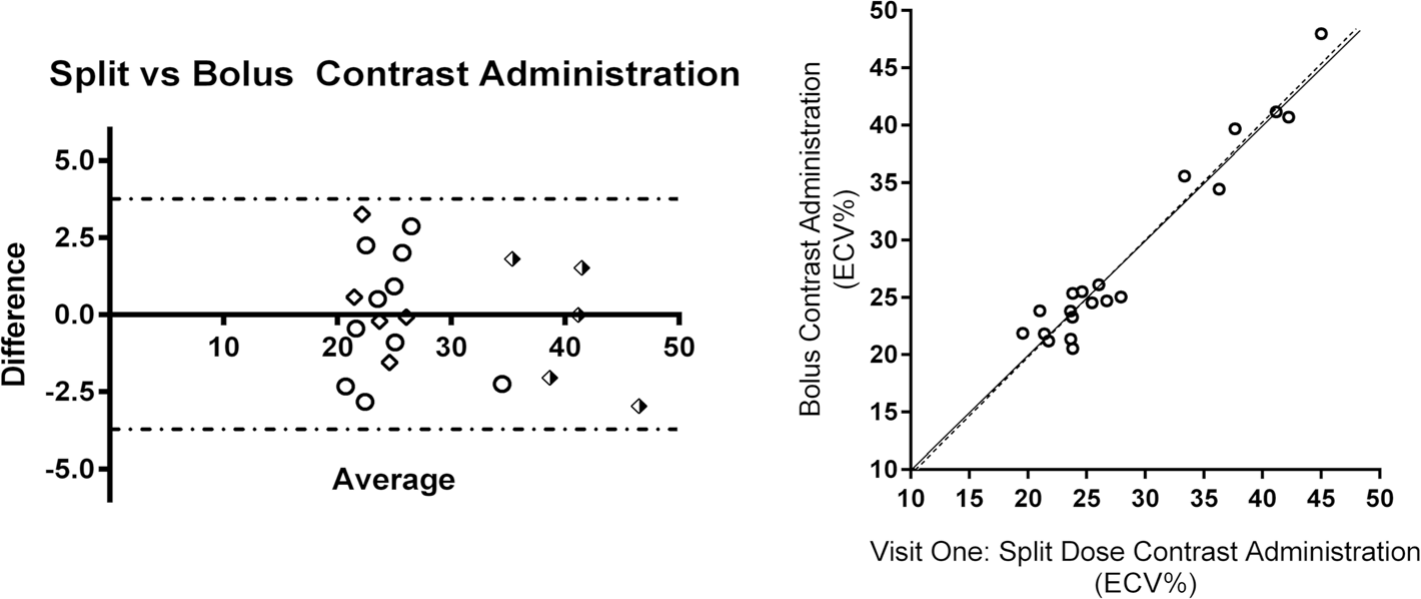
**Bland Altman plot of agreement between ECV estimated using single bolus and split-dose contrast administrations in patients and healthy volunteers (bias 0.009, 95% CI −3.754 to 3.772, r** ^**2**^ **= 0.973, p = 0.001).** Bland Altman: Shaded diamond: *Pathological Myocardium*; Diamond: *Remote myocardium*; Circle: *Healthy Volunteer*. Correlation Plot: Circle: *All study subjects*.

There was a strong positive correlation between ECV calculated following single or split bolus contrast administration in healthy volunteers, as well as abnormal and remote myocardium in patients (coefficient of variability 5.04%; bias 0.009, 95% CI −3.754 to 3.772, r^2^ = 0.973, p = 0.001)). Bland-Altman plot of the data set can be seen in Figure 1.

**Figure 2:**
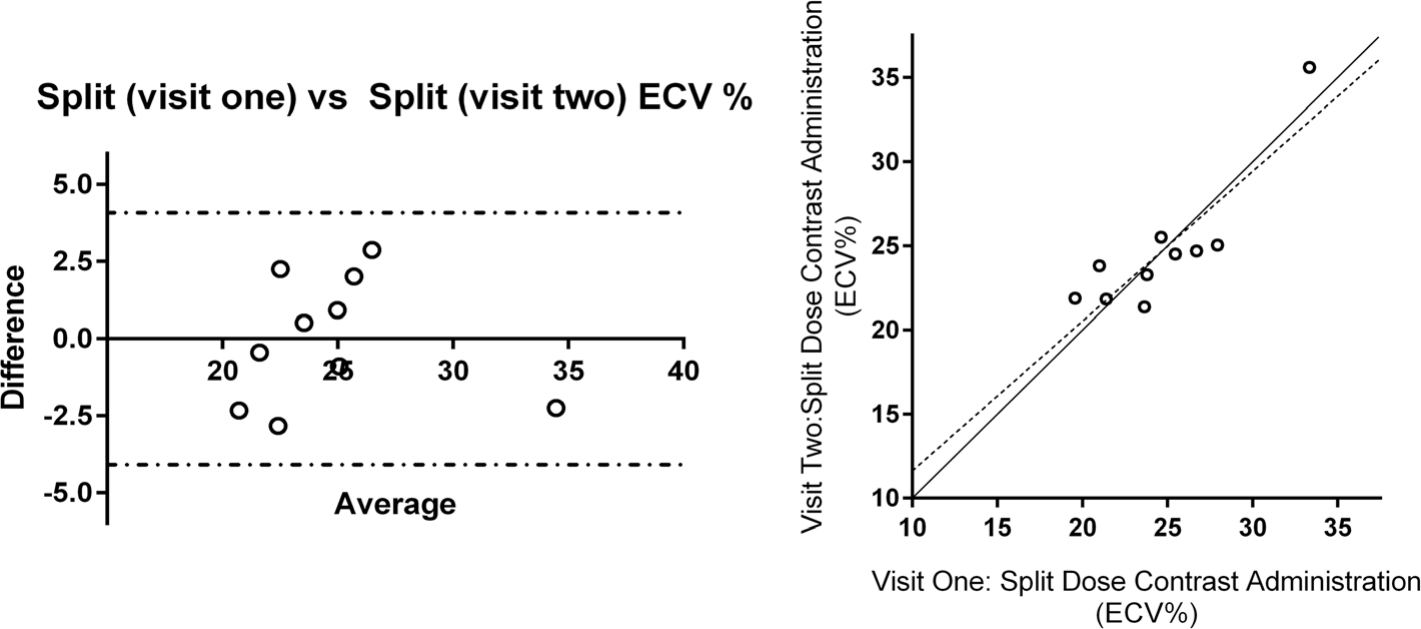
**Bland Altman plot of agreement of ECV estimates between visit 1 and 2 using split-dose administration in healthy volunteers (bias −0.018, 95% CI −4.045 to 4.009, r** ^**2**^ **= 0.757, p = 0.001).**

Inter-study agreement for ECV calculation with split dose administration visits was good in the 10 volunteers studied (coefficient of variability 5.67%, r^2^ = 0.766, p < 0.001). Bland-Altman plot can be seen in Figure 2 (bias −0.018, 95% CI −4.045 to 4.009).

## Discussion

The insights that T1 mapping offers into tissue composition are increasingly applied as a research tool. T1 mapping is also being integrated into clinical protocols, particularly in the investigation of unexplained left ventricular hypertrophy. Consequently, comprehensive CMR protocols that interrogate not only cardiac structure, function and perfusion but also tissue composition in one protocol have great potential clinical value.

Previous studies have shown that ECV calculated using either an infusion or bolus of contrast agent [12] is reproducible, and correlates well with fibrosis measured on myocardial biopsy specimens [19]. However until this time it was not known if split dose contrast administration, as used in adenosine stress perfusion protocols affects, ECV estimation and how this correlates with previously validated methods.

Given that reliable ECV calculation requires steady state of contrast agent concentration, split dose administration may have given rise to different estimates against single bolus administration or a continuous contrast infusion. Any such differences would have prevented the application of established normal ranges that have been published over recent years to subjects undergoing stress perfusion protocols [8,9,19-21]. It was also conceivable that vasodilator effects of adenosine stress may have led to different contrast distribution with the myocardium and peripheral tissues. Persistent vasodilation at the time of the second MOLLI, as a consequence of adenosine administration would lead to a genuine increase of ECV due to increased capillary plasma volume. However the vasodilatory effects of adenosine are both transitory and short-lived [22] and have passed by the time of rest perfusion acquisition and following that, the second MOLLI.

This study has now shown that ECV estimation with split dose contrast administration as part of a stress/rest perfusion CMR protocol agrees well with bolus administration in healthy volunteers. Reproducibility and inter-study agreement was good for split dose ECV calculations and in line with that previously published for ECV calculation following bolus contrast administration [12].

Previously published data suggested that bolus contrast administration may underestimate ECV at values of >40% [12]. However in this study we have examined 5 patients with extensive LGE enhancement and grossly elevated ECV due to chronic myocardial infarction and cardiomyopathy and found equivalence between the techniques. This suggests that ECV calculation is reliable across a range of values using either method of contrast administration.

### Limitations

This study was performed in a limited number of healthy volunteers and patients. It has previously been shown that at fifteen minutes contrast equilibrium may not have been reached for post contrast T1 mapping, especially in individuals with higher ECVs. [12] This study has attempted to address this point specifically, however no patients with the most elevated ECVs (eg cardiac amyloidosis) were studied, due in part to the demands of returning for a non-clinically indicated research CMR study. In spite of this we have shown equivalence of these techniques in ECV calculation up to an ECV of 48%.

This study investigated one particular MOLLI acquisition scheme which is consistent with international recommendations. However, other methods have been published and as yet there is no firm MOLLI scheme recommendation. It is difficult to select the ideal T1 mapping sequence as different sequences may perform differently depending upon the T1 of the tissue studied. Consequently the demonstration of the inter-study reproducibility of locally adopted sequences, in line with SCMR guidance, is important.

## Conclusion

Split dose contrast T1 mapping, in keeping with a stress perfusion protocol, is reproducible and agrees with bolus contrast administration. This suggests ECV measurement maybe incorporated into stress perfusion protocols in both clinical and research CMR.
